# Influence of Molecular Weight and Lithium Bis(trifluoromethanesulfonyl)imide on the Thermal Processability of Poly(ethylene oxide) for Solid-State Electrolytes

**DOI:** 10.3390/polym15163375

**Published:** 2023-08-11

**Authors:** Katharina Platen, Frederieke Langer, Roland Bayer, Robert Hollmann, Julian Schwenzel, Matthias Busse

**Affiliations:** 1 Fraunhofer Institute for Manufacturing Technology and Advanced Materials (IFAM), Lilienthalplatz 1, 38108 Braunschweig, Germany; 2 DDP Specialty Products Germany GmbH & Co. KG, Business Unit Pharma Solutions/Health, International Flavors & Fragrances Inc. (IFF), August-Wolff-Straße 13, 29699 Walsrode-Bomlitz, Germany; 3 Fraunhofer Institute for Manufacturing Technology and Advanced Materials (IFAM), Wiener Straße 12, 28359 Bremen, Germany

**Keywords:** PEO, LiTFSI, thermal processing, production, kneading, extrusion, all-solid-state batteries

## Abstract

New energy systems such as all-solid-state battery (ASSB) technology are becoming increasingly important today. Recently, researchers have been investigating the transition from the lab-scale production of ASSB components to a larger scale. Poly(ethylene oxide) (PEO) is a promising candidate for the large-scale production of polymer-based solid electrolytes (SPEs) because it offers many processing options. Hence, in this work, the thermal processing route for a PEO-Lithium bis(trifluoromethylsulfonyl)imide (LiTFSI) SPE in the ratio of 20:1 (EO:Li) is investigated using kneading experiments. Here, we clearly show the sensitivity of PEO during thermal processing, especially for high-molecular-weight PEO (M_w_ = 600,000 g mol^−1^). LiTFSI acts as a plasticizer for low-molecular-weight PEO (M_w_ = 100,000 g mol^−1^), while it amplifies the degradation of high-molecular-weight PEO. Further, LiTFSI affects the thermal properties of PEO and its crystallinity. This leads to a higher chain mobility in the polymer matrix, which improves the flowability. In addition, the spherulite size of the produced PEO electrolytes differs from the molecular weight. This work demonstrates that low-molecular-weight PEO is more suitable for thermal processing as a solid electrolyte due to the process stability. High-molecular-weight PEO, especially, is strongly influenced by the process settings and LiTFSI.

## 1. Introduction

Polymeric materials represent, alongside oxidic and sulfidic materials, one of the three main material classes for use as solid-state electrolytes for all-solid-state batteries (ASSB) [1,2,3]. The specialty of these materials is to provide a path for ion migration from electrodes and at the same time to separate the electrodes to avoid short circuits [4]. In general, the replacement of an organic liquid electrolyte with an ion-conducting solid that is mechanically stable towards Li dendrite formation [5,6,7] enables the use of metallic lithium as an anode. This leads to a higher capacity and cell voltage than conventional batteries. In addition, the use of nonvolatile and less flammable materials such as polymers improves the safety of such a battery [8]. Polymers, which provide a malleable and elastic nature, are able to compensate for large volume changes in the active materials in the electrodes during discharging and charging [9].

While promising materials have been identified and extensively characterized with regard to their chemical, structural, and electrochemical properties, the material availability in the case of oxide and sulfide materials and the knowledge of scaled production routes for all types of separators still hinder the commercialization of ASSBs.

Two main aspects for the introduction of industrial production of solid electrolytes are the manufacturability and their cost [10]. Both aspects need to be considered in order to scale up the process. Solid polymer electrolytes (SPEs) can be produced in many ways like solution casting [4], spin coating [4], hot pressing [4,11,12,13], tape casting [4], screen printing [4], dip coating [4], and extrusion [14,15,16]. One of the most common manufacturing methods at the laboratory scale for polymer electrolytes is solution casting [17,18,19,20,21,22,23]. The drawbacks of this method are as follows: Firstly, there are a large number of necessary processing steps required, i.e., the premixing of the solid components, the preparation of a homogeneous slurry in which the components are fully dissolved, and a drying step to remove the solvent. Secondly, residual solvents can have a negative effect on the electrochemical performance of the polymer electrolyte [24]. Due to these drawbacks, a manufacturing method without the need of any solvents is preferable.

Possible process chains for solid electrolytes and batteries are described by Schnell et al. [10]. Here, the authors highlight melt processing as a mixing step, where conductive salt is dissolved in a polymer melt without the need of solvents. Thermoplastic polymers offer the possibility of such a melt process for the production of polymer-based electrolytes, e.g., the extrusion process. This continuous process holds potential for easily scaling up the processing of such SPEs from the laboratory scale to industrial mass production. A common thermoplastic material for research works with regard to electrochemical properties and performance for use as a polymer electrolyte is poly(ethylene oxide) (PEO) [25,26,27,28,29,30,31,32]. PEO has a melting temperature (T_m_) around 65 °C, depending on its molecular weight [33]. Furthermore, it can dissolve alkali salts such as Lithium bis(trifluoromethanesulfonyl)imide (LiTFSI), resulting in ionic conductivities of (4 ± 2) × 10^−4^ S cm^−1^ at 60 °C. The ionic conductivity does not depend on the molecular weight above a M_w_-value of 2000 [27]. Thus, the focus of such a well-studied polymer electrolyte must now be to produce it on a larger scale while maintaining its properties to be competitive with conventional LIBs. In addition, the effect of process parameters on the properties of PEO-based solid electrolytes needs to be thoroughly investigated. It is known that LiTFSI has an effect on the thermal properties and crystallization behaviour of PEO [34,35,36,37]. In the case of thermal processing, LiTFSI acts as a plasticizer that enhances the flowability of the polymeric material.

The thermal processing route of PEO and of SPEs with PEO as a polymer component has become increasingly important in recent years [16,38,39,40]. Studies so far have focused on the thermal stability of high-molecular-weight PEO (M_w_ = 600 k–4 M) during processing as polymer electrolyte or as a polymer-based composite cathode using a kneader or extruder and the material stability during storage. High-molecular-weight PEO shows sensitivity to process parameters, e.g., high temperature or kneading speed.

In this study, we focus on the process behavior of PEO during thermal processing with a laboratory kneader in order to predict the suitable process parameters for extrusion processes with the obtained results. We determine the influence of process parameters on PEO and PEO-based electrolytes. In addition, we investigate the influence of molecular weight and the addition of LiTFSI on the processability of PEO. Due to the sensitivity of PEO during processing, the process parameters are optimized to lower device temperatures and kneading speeds. For characterization, we determine the thermal properties of PEO and electrolytes, as well as the molecular weight after kneading and homogeneity, using microscopic characterization.

## 2. Materials and Methods

### 2.1. Materials

For the investigation of the thermal process behavior of polymer-based solid-state electrolytes, PEO (M_w_(PEO) = 100,000 g mol^−1^ and 600,000 g mol^−1^, IFF, New York, NY, USA) was used as a thermoplastic polymer and LiTFSI (S001A, Solvionic, Toulouse, France) as a conductive salt. To determine the influence of the molecular weight of the polymer on the thermal process stability and to ensure that the ionic conductivity of the produced electrolytes behaved independently of the molecular weight, a molecular weight >2 × 10^3^ g mol^−1^ was chosen for this work. Therefore, PEO100k (Polyox WSR N10) and PEO600k (Polyox WSR 205) were used for low-M_w_ and high-M_w_ PEO, respectively.

Since the coordination of Li ions occurs through the ether bridge of the polymer, the molar ratio between the ether group of the PEO monomer (EO) and the Li ion (Li) was used for the mixing ratio of the polymer electrolyte [41]. It is stated as EO:Li and was calculated as follows:

(1) $$\frac{n_{\mathrm{EO}}}{n_{\mathrm{Li}}}=\frac{m_{\mathrm{EO}}\times M_{\mathrm{LiTFSI}}}{m_{\mathrm{LiTFSI}}\times M_{\mathrm{EO}}}$$

The solid electrolytes investigated in this work had a constant molar ratio of 20:1 (EO:Li) since this composition exhibits relatively high ionic conductivity at elevated temperatures [42]. The corresponding weight ratio for this mixture is w_PEO_/w_LiTFSI_ = 0.75/0.25. Further, since the stickiness increases with increasing LiTFSI concentration [36,43], the chosen composition simplifies the handling during the thermal processing of the polymer electrolyte. To avoid any influence of residual water on the process behavior of the polymer electrolyte, all components were dried in a vacuum for 12 h before testing. The drying temperatures were 50 °C and 120 °C for PEO and LiTFSI, respectively.

### 2.2. DSC Measurements

For DSC measurement, a Discovery DSC (TA Instruments) was used. Aluminum crucibles were filled with PEO powder and a mixture of PEO powder and LiTFSI powder and sealed under an inert atmosphere. To melt the polymer and to dissolve the LiTFSI in the polymer matrix, the components were first heated up to 120 °C with a heating ramp of 10 °C min^−1^, eliminating the thermal history. In a second heating run, from −80 to 180 °C with a heating ramp of 10 °C min^−1^, the thermal properties, glass transition temperature (T_g_), melting temperature (T_m_), and crystallinity (K) were determined for neat-PEO and (PEO)_20_LiTFSI electrolytes.

### 2.3. Kneading Experiments

To characterize the thermal processing behavior of PEO and (PEO)_20_LiTFSI electrolytes, a Plasti-Corder/Lab-Station (Brabender, Duisburg, Germany) with kneading equipment (sample volume of 50 mL and two sigma shovels) was used. During processing, torque and mass temperature were measured as a function of the applied device temperature and kneading speed. The kneading experiments were performed at a device temperature of 90 and 100 °C and with kneading speeds of 5, 10, 15, and 20 min^−1^ for neat PEO and 5 and 20 min^−1^ for (PEO)_20_LiTFSI electrolytes. To investigate the influence of LiTFSI on the thermal processing behavior, PEO was first melted under process conditions. After the polymer was completely and homogeneous melted, LiTFSI was added to the polymer melt 20 min after the start of the experiment, as determined from the neat-PEO experiments.

### 2.4. Digital Microscopy Observations

For a microscopic observation of the kneaded samples, a VHX7000 digital microscope (Keyence, Osaka, Japan) with a polarizing filter was used. For sample preparation, the kneaded (PEO)_20_LiTFSI-samples were hot-pressed to different layer thicknesses in an argon-filled glovebox. For hot-pressing, care was taken so that the applied pressing temperature was the same as under kneading conditions. To characterize the homogeneity of the kneaded (PEO)_20_LiTFSI electrolytes, the samples were pressed to a layer thickness of 100 µm. For an observation of the crystal growth of the polymeric material, the samples were hot-pressed to a layer thickness of 10 µm. Observations were made with incident light and with transmitted light using a polarizing filter, respectively. The kneaded neat PEO samples were examined microscopically without any preparation. The spherulite size was determined by measuring the size of more than 10 spherulites from the center to the edge.

### 2.5. Scanning Electron Microscopy and EDX Analysis

To support the digital microscopy observations concerning the material stability after kneading, the surface morphologies of the kneaded PEO samples were obtained using scanning electron microscopy (SEM, Phenom^TM^ Accurion proX, Waltham, MA, USA) in an argon-filled glovebox operating at 10 kV. All samples were sputtered with Pt/Pd prior to microscopy. EDX analysis was performed to investigate the salt distribution within the kneaded (PEO)_20_LiTFSI samples operated at 15 kV. The LiTFSI distribution was determined by the detection of fluorine (F) and sulfur (S).

### 2.6. GPC Measurments

Gel permeation chromatography (GPC) measurements were performed to determine the molecular weight of the kneaded PEO and (PEO)_20_LiTFSI samples. All samples were dissolved in a 0.05% (aq.) NaN_3_ solution. In this work, the number average molecular weight, M_n_, and the polydispersity are examined to evaluate the polymer stability during processing. Polydispersity is a measure of the width of a molecular mass distribution and is calculated as follows:

(2) $$d=\frac{\;M_{w}}{\;M_{n}}$$

## 3. Results and Discussion

### 3.1. DSC Measurements

**Figure 1:**
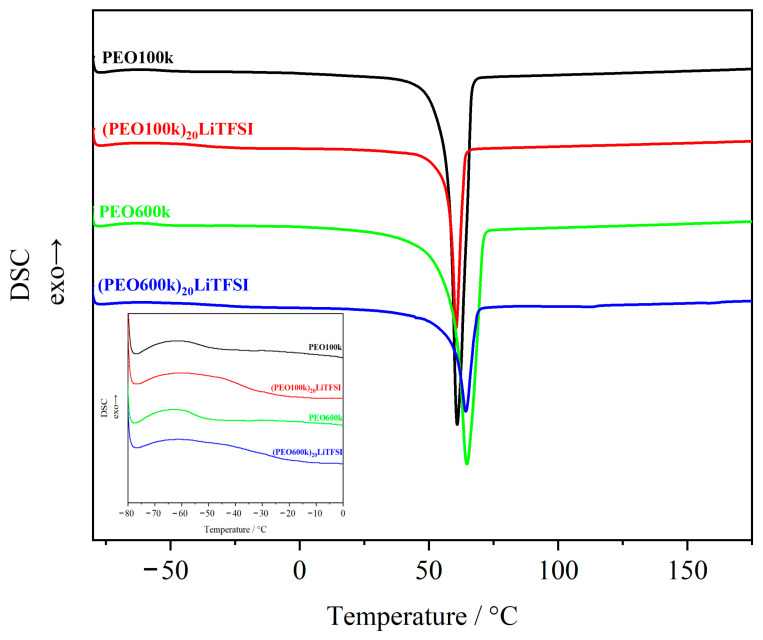
DSC curves of neat PEO and PEO_20_LiTFSI electrolytes with different molecular weights; determination of the glass transition temperature (T_g_), melting temperature (T_m_), and crystallinity (K) from the 2nd heating run to avoid any effects of the thermal history.

Figure 1 shows the DSC curves of the second heating run for PEO and (PEO)_20_LiTFSI electrolytes. The extracted thermal properties are listed in Table 1. The thermal properties of PEO showed a dependence on the molecular weight as well as on the addition of LiTFSI. The thermal property most affected by the molecular weight was the melting temperature (T_m_) of the crystalline portion of the polymer. T_m_ was determined by the maximum of the endothermic peak. As the molecular weight increased, T_m_ shifted to higher temperatures. PEO600k had its T_m_ at 65 °C, which is 4 °C higher than the T_m_ of PEO100k (T_m_ = 61 °C). The effect of the molecular weight on the thermal properties was in a good agreement with previous works [39,44,45,46]. CROWLEY and coworkers found that low-molecular-weight PEO has a higher proportion of small crystals by studying PEO with different molecular weights (M_w_ = 100,000 and M_w_ = 1,000,000) [39]. Therefore, smaller crystals require less energy for melting than larger crystals in PEO with a higher molecular weight. In general, for semicrystalline polymers, it can be estimated that the crystal growth rate decreases with increasing molecular weight [45]. Furthermore, VRANDEČIĆ et al. described the crystalline phase of PEO by comparing different molecular weights (M_w_ = 100,000–5,000,000). The higher the molecular weight of PEO, the lower the segmental mobility and convenient geometrical alignment within the polymer chain [46]. All these points underline the shift in T_m_ to a higher temperature with a higher M_w_. For thermal processing, this minor shift in T_m_ of 4 °C does not affect the process window of PEO. Thus, the same process temperatures can be selected for both molecular weights.

The degree of crystallinity was calculated from the ratio of the experimentally determined melting enthalpy to that of a 100% crystalline PEO, as follows:

(3) $$K=\frac{∆H_{m(\exp.)}}{∆H_{\left(100\%\;\mathrm{cryst}.\;\mathrm{PEO}\right)}}$$

with ΔH_(100% cryst. PEO)_ = 203 J g^−1^ [47]. The crystallinity of the neat PEO was not affected by the molecular weight (Table 1). A possible reason may be the slow crystallization kinetics of PEO [48], so the spherulites do not grow to their initial size during the cooling run.

Compared to the molecular weight, the addition of LiTFSI showed a minor effect on T_m_. Since the thermal properties of polymer electrolytes have already been investigated in other works, i.e., by MARZANTOWICZ and coworkers, EDMAN et al., and LASCAUD et al., the effect of LiTFSI and other Li salts on the thermal properties of PEO is well understood [34,35,47,49,50,51]. The authors focused on the influence of LiTFSI on the recrystallization behavior of PEO and found that the recrystallization kinetics decrease with a higher amount of Li salt. These observations were made in the second heating run of a quenched sample, where a shift in or even a disappearance of T_m_ was observed. However, the determined T_m_ of salt containing PEO was in good agreement with the results of this work [46,50]. In addition, LiTFSI obviously reduces the peak height, which directly affects the crystallinity of the polymer electrolyte. Furthermore, for (PEO)_n_LiTFSI electrolytes with the TFSI anion as a crystal inhibitor, nucleation is the limiting step for crystallization [35]. Crystallinity decreased from 70% to 28% for both molecular weights (Table 1).

Since the crystallinity (K) did not change as a function of M_w_, the glass transition temperature (T_g_) also remained unchanged for different M_w_ values. T_g_ was determined as the midpoint of the endothermic step in Figure 1. This is in good agreement with the observation made by VRANDEČIĆ and coworkers in their work [46]. T_g_ and K were not affected by the molecular weight. In contrast to the molecular weight, the addition of LiTFSI mainly affected the amorphous region of the polymer. The addition of LiTFSI led to a lower chain mobility in the polymeric matrix, which shifted T_g_ to higher temperatures (T_g((PEO100k)20LiTFSI)_ = −40 °C and T_g((PEO600k)20LiTFSI)_ = −34 °C). The Li-cation coordinates oxygen-atoms from different PEO-chains resulting in a three-dimensional physical network.

### 3.2. Kneading Experiments

#### 3.2.1. Effect of Process Parameters and Molecular Weight on the Thermal Kneading Behavior of PEO

The DSC results show that PEO100k and PEO600k can be processed under the same process conditions. To ensure that the polymer was completely melted and had a good flowability during the process, process temperatures of 90 and 100 °C were selected for this work.

**Figure 2:**
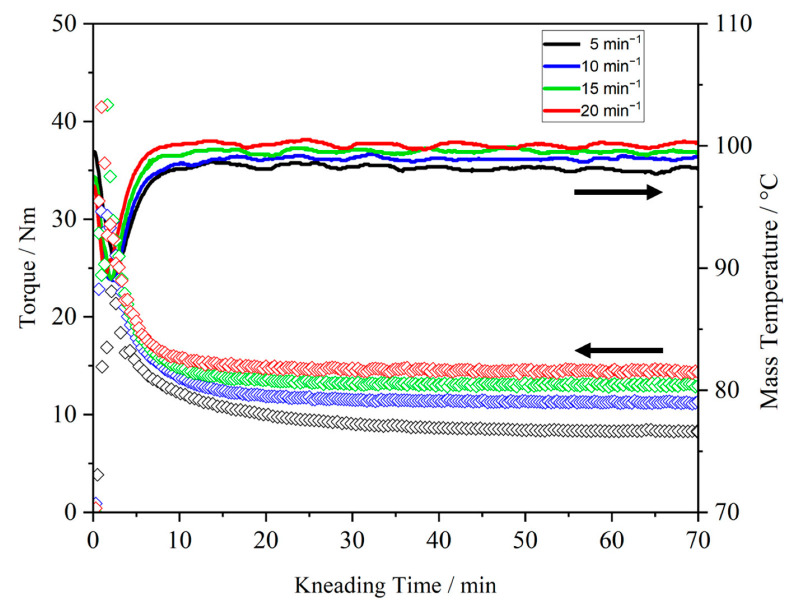
Kneading curves of neat PEO100k at a process temperature of 100 °C and with different kneading speeds (5–20 min^−1^).

First, the kneading behavior of neat PEO was determined as a function of the process parameters and its molecular weight. Figure 2 shows the kneading curve of PEO100k. The applied kneading temperature was 100 °C and the kneading speed varied from 5 to 20 min^−1^. During the first 20 min, the melting process of the polymer can be observed, which is characterized by the decrease in torque. The completion of the melting process was determined by the offset of the peak maximum of the torque. The higher the applied kneading speed, the faster the polymer was completely melted (offset for 5 min^−1^: 17 min, 9 N m; offset for 20 min^−1^: 4 min, 15 N m).

The kneading behavior of PEO was influenced by the process parameters used. The higher the kneading speed, the higher the measured torque and mass temperature. The measured torque of the kneading experiment with 20 min^−1^ (14.5 ± 0.2 N m) was about 39% higher than for 5 min^−1^ (8.8 ± 0.4 N m). Higher kneading speeds resulting in higher torque values were the result of entanglement of the polymer chains during kneading; while the polymer chains had more time to rearrange at a kneading speed of 5 min^−1^, the shear effect at a kneading speed of 20 min^−1^ gave the polymer chains less time to relax. The presence of different levels of shear effects within the material was characterized by the difference in mass temperature. Increased shear effects caused the self-heating of the material, resulting in a higher mass temperature. The same dependencies of kneading speed on the kneading behavior could be observed at a kneading temperature of 90 °C. For the extrusion of PEO, a process temperature of 90 °C was preferable due to the lower heat impact on the polymer.

The determination of melt completion can be used as the minimum residence time for PEO in the extruder barrel.

**Figure 3:**
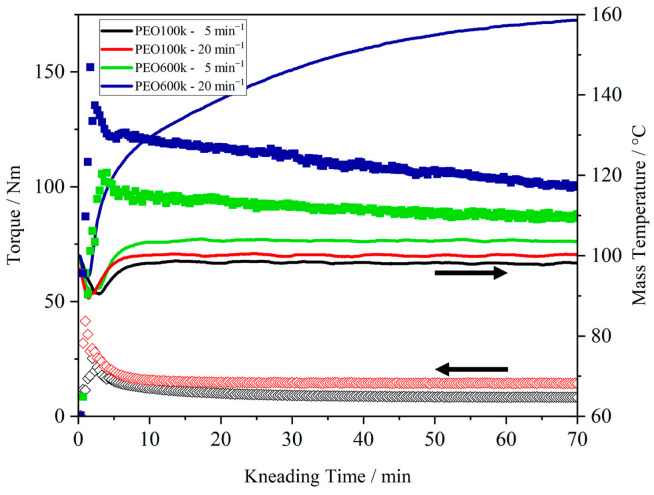
Comparison of kneading curve of PEO with different molecular weight (100 k and 600 k) at a process temperature of 100 °C and with kneading speeds of 5 and 20 min^−1^.

Figure 3 shows the dependence of the molecular weight on the kneading behavior by comparing the kneading behavior of PEO100k with PEO600k at a kneading temperature of 100 °C and kneading speeds of 5 and 20 min^−1^. A strong influence of the molecular weight on the resulting torque was evident. The use of PEO600k resulted in significantly higher torque values compared to PEO100k over the entire experiment time (t = 50 min, 5 min^−1^: PEO100k = 8 N m, PEO600k = 89 N m; 20 min^−1^: PEO100k = 15 N m, PEO600k = 107 N m). Furthermore, even after 20 min, when a stable melt was obtained for PEO100k, PEO600k performed less stably, characterized by a negative slope in the torque (5 min^−1^: −0.15; 20 min^−1^: −0.34). The mass temperature of PEO600k, especially at high kneading speed, increased to 160 °C. With higher kneading speeds (20 min^−1^), the shear effect within the material due to longer chain lengths led to the self-heating of the polymer and, thus, to a higher mass temperature over time. The steady decrease in torque and the self-heating effect indicate the degradation of PEO600k at a processing temperature of 100 °C. PEO is a very sensitive material to thermal, oxidative, and mechanical degradation. The primary mechanism of a degradation process is chain scission, forming chains of lower molecular weight [39]. Since no decomposition reaction was observed during the DSC measurements (Figure 1), the degradation of PEO600k during the kneading experiments was not only dependent on the device temperature. Hence, the main driving force for the degradation of PEO600k was a combination of the heat generated by the device temperature, the self-heating effect of the long chains of PEO600k, and the mechanical force induced by the screw speed.

The kneading behavior of PEO600k for different kneading speeds, device temperatures, and filling degrees is well described elsewhere [16]. Although the kneading conditions here were chosen considering the sensitivity of the material, the degradation of PEO600k occurred, as described earlier. Furthermore, the self-heating effect and degradation of PEO600k even occurred at 90 °C. The results of the kneading experiments show that PEO100k behaved more stably for the applied process temperatures and kneading speeds, and it is therefore more suitable for thermal processing.

#### 3.2.2. Effect of the Addition of LiTFSI on the Thermal Kneading Behavior of PEO

**Figure 4:**
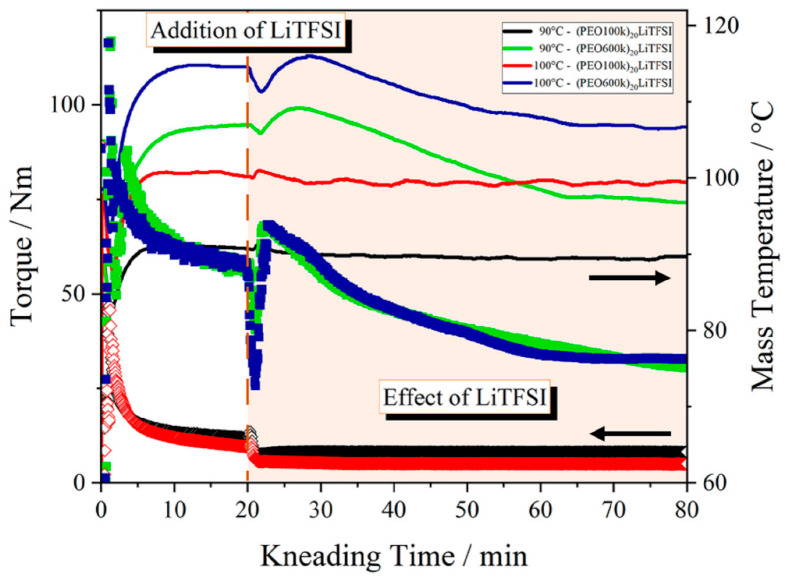
Effect of LiTFSI on the thermal kneading behavior of PEO with different molecular weights at a process temperature of 90 and 100 °C and with kneading speed of 20 min^−1^.

For the thermal processing route of polymer-based solid electrolytes, it is important to know how the lithium salt affects the thermal properties and the processing window of the polymeric material. Figure 4 shows the exemplary kneading curves of (PEO100k)_20_LiTFSI and (PEO600k)_20_LiTFSI electrolytes for a kneading speed of 20 min^−1^ and process temperatures of 90 and 100 °C. The kneading speed of 20 min^−1^ was chosen for representation because it represents the harshest process conditions within this work. LiTFSI was added to the process after 20 min of kneading PEO because the previous experiments confirmed that PEO is completely melted at this point. The kneading chamber was opened to add LiTFSI. The addition of LiTFSI was marked as the starting point for the mixing of the polymer with LiTFSI. For both molecular weights, we can see an effect due to the addition of LiTFSI.

The kneading curves of PEO100k show a step-like drop in torque after the addition of LiTFSI to the process for both temperatures. The kneading curve for 100 °C shows a loss of torque of 5.5 N m. After the torque drop, it remained constant for the remaining kneading time. The loss of torque indicates that LiTFSI acts as a plasticizer that is mixed and dissolved in the polymer matrix. The addition of LiTFSI only had a negligible effect on the mass temperature. Further, the TFSI anion was also embedded between the polymer chains. This led to a higher chain mobility and higher flowability in the polymer during thermal processing.

Compared to the kneading curve for PEO100k at 100 °C, the kneading curve for PEO600k at 100 °C does not show a step-like drop in torque. Instead, a minimum in the torque was observed due to the opening of the kneading chamber. The effect of opening the kneading chamber was higher for PEO600k than for PEO100k. After the filling of LiTFSI and closing the kneading chamber, the torque increased slightly compared to the values before opening the chamber due to the cooling effect of LiTFSI and the increased volume inside the chamber. However, the torque then decreased continuously by almost 50% within 55 min. In contrast to the PEO100k sample, where LiTFSI acted as a plasticizer, it seems that LiTFSI accelerated the degradation process of PEO600k. The characteristic negative slope of the torque curves increased by 51% for the 90 °C curve and by 40% for the 100 °C curve.

The mass temperature curve of (PEO600k)_20_LiTFSI shows a decrease in mass temperature due to the addition of the colder LiTFSI powder. In contrast to the self-heating and shear effect as shown in Figure 3, the mass temperature of (PEO600k)_20_LiTFSI decreased over the kneading time. Both the decrease in mass temperature and the decrease in torque indicate that the plasticizing effect of LiTFSI is overlapped by the degradation process of PEO600k. The degradation overrides the plasticizing effect, as it is assumed that the decrease in mass temperature close to the device temperature is due to smaller chains resulting in less shearing of the polymer chains.

The kneading curves for 90 °C show the same dependencies of LiTFSI on the processability of PEO100k and PEO600k. In particular, the kneading curves of PEO100k for both temperatures show a loss of torque of 4.5 N m for 90 °C and 5.5 N m at 100 °C. The loss of torque is caused by both the addition of LiTFSI and the applied kneading temperature. Since the difference in the loss of torque was only 1 N m, LiTFSI, acting as plasticizer, had the greatest effect on the torque.

The kneading experiments show that the processing parameters of PEO100k are stable after the addition of LiTFSI. Hence, it is more suitable for use as an electrolyte material than PEO600k. Since there is no effect of temperature and kneading speed on the polymer stability, both the investigated temperature and kneading speed are suitable for the thermal process. However, low temperatures and kneading speeds are preferable to keep process stresses in the form of shear effects and elevated mass temperatures as low as possible.

### 3.3. Digital Microscopy Observations

**Figure 5:**
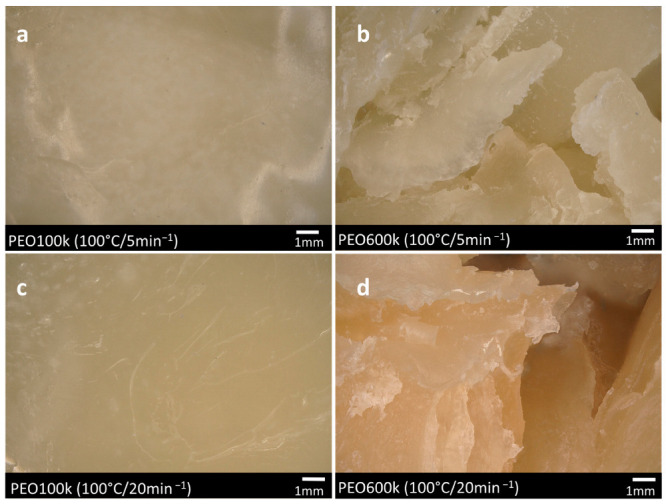
Microscopic images of kneaded PEO100k (**a**,**c**) and PEO600k (**b**,**d**).

Figure 5 shows microscopic images of the kneaded PEO100k and PEO600k samples that were kneaded at 100 °C with kneading speeds of 5 and 20 min^−1^, as described earlier in this work. The PEO100k samples show a smooth surface and no color change for both kneading speeds. The shiny surface reflects the incident light, the images are low in contrast, and individual particles or shapes cannot be distinguished. During the extraction of the PEO100k samples after kneading, the polymer melt appeared waxy and sticky. Therefore, it was assumed that the polymer structure remained unchanged regardless of the process parameters. In contrast, PEO600k shows a broken surface structure, especially for the samples kneaded at 20 min^−1^. In contrast to PEO100k, PEO600k appeared dry and brittle during extraction. The images depict a multitude of fractured surfaces with irregular edges. These microscopic images also indicate the described degradation process of the polymer due to self-heating and shear effects. Here, the degradation was characterized by a change in color. The discoloration intensified with higher kneading speeds for PEO600k.

**Figure 6:**
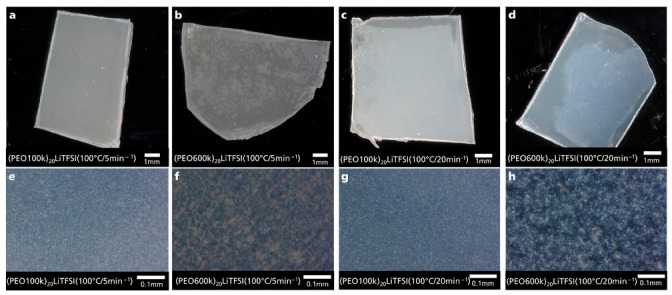
Microscopic images of kneaded polymer electrolytes: (**a**,**b**,**e**,**f**): (PEO100k)_20_LiTFSI and (PEO600k)_20_LiTFSI kneaded at 100 °C with a kneading speed of 5 min^−1^, hot-pressed for microscopic investigation to a layer thickness of 100 µm and 10 µm, respectively; (**c**,**d**,**g**,**h**): (PEO100k)_20_LiTFSI and (PEO600k)_20_LiTFSI kneaded at 100 °C with a kneading speed of 20 min^−1^, hot-pressed for microscopic investigation to a layer thickness of 100 µm and 10 µm, respectively.

Further microscopic observations were performed to investigate the homogeneity of the kneaded (PEO)_20_LiTFSI electrolytes and to determine the effect of LiTFSI on the crystal growth within the polymer matrix due to its importance and influence on the ionic conductivity. Figure 6 shows microscopic images of (PEO100k)_20_LiTFSI electrolytes and (PEO600k)_20_LiTFSI electrolytes kneaded at a process temperature of 100 °C and kneading speeds of 5 and 20 min^−1^. The kneaded samples were hot-pressed to 100 µm and 10 µm thickness, respectively, to examine the homogeneity and crystal growth. The images a–d, using incident light, show the LiTFSI distribution in the polymer matrix depending on the molecular weight of PEO and the applied kneading speed. It seems that for both molecular weights, LiTFSI was completely dissolved in the PEO matrix as no residues of LiTFSI particles were detected. However, differences in the surface of the samples are noticeable. For example, the kneaded (PEO600k)_20_LiTFSI sample (Figure 6b) appears more irregular in its surface compared to the kneaded (PEO100k)_20_LiTFSI sample (Figure 6a). Overall, the morphology of the pressed samples of (PEO100k)_20_LiTFSI electrolytes appears more homogeneous than PEO600k. Figure 6e–h depict the influence of molecular weight on crystal growth. The spherulite size was determined by measuring the distance from the center to the edge. (PEO100k)_20_LiTFSI shows spherulites with a size < 10 µm (5 min^−1^: 8 ± 1 µm; 20 min^−1^: 6 ± 1 µm) independent of the kneading speed. Instead, (PEO600k)_20_LiTFSI shows larger spherulites (5 min^−1^: 30 ± 9 µm; 20 min^−1^: 30 ± 8 µm) for both kneading speeds. Crystal growth appears to have been unaffected by the applied kneading speed. In addition, the DSC results in this study show that T_m_ increases with molecular weight, thus requiring a higher temperature to melt the polymer crystals. From the correlation of spherulite size and melt temperature with molecular weight, the molecular weight plays a key role in crystal growth. These findings are in accordance with previous studies [39,45].

### 3.4. Scanning Electron Microscopy and EDX Analysis

**Figure 7:**
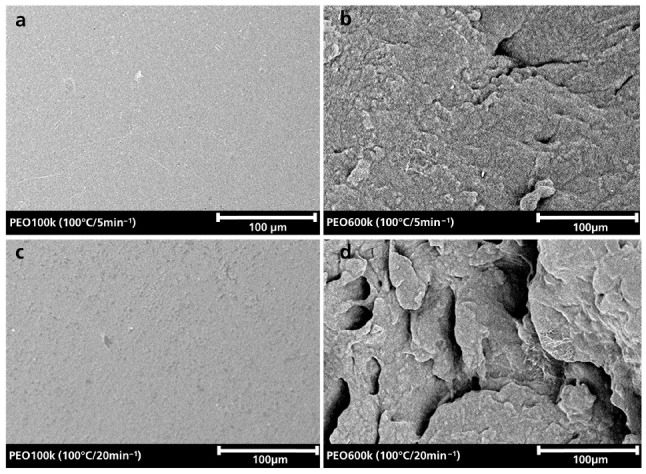
SEM images of kneaded PEO100k (**a**,**c**) and PEO600k (**b**,**d**).

Figure 7 shows SEM images of PEO100k and PEO600k samples that were kneaded at 100 °C at kneading speeds of 5 and 20 min^−1^. The PEO100k sample kneaded at 5 min^−1^ (Figure 7a) shows a smooth surface, as already described above. The sample kneaded at 20 min^−1^ (Figure 7b) shows a slight change in surface roughness. Both kneaded PEO600k samples (Figure 7c,d) show a broken surface structure that becomes stronger with increasing kneading speed. Compared to PEO100k, these samples were more damaged after kneading, which supports the assumption of degradation of the polymer that was made from the digital microscopy images.

**Figure 8:**
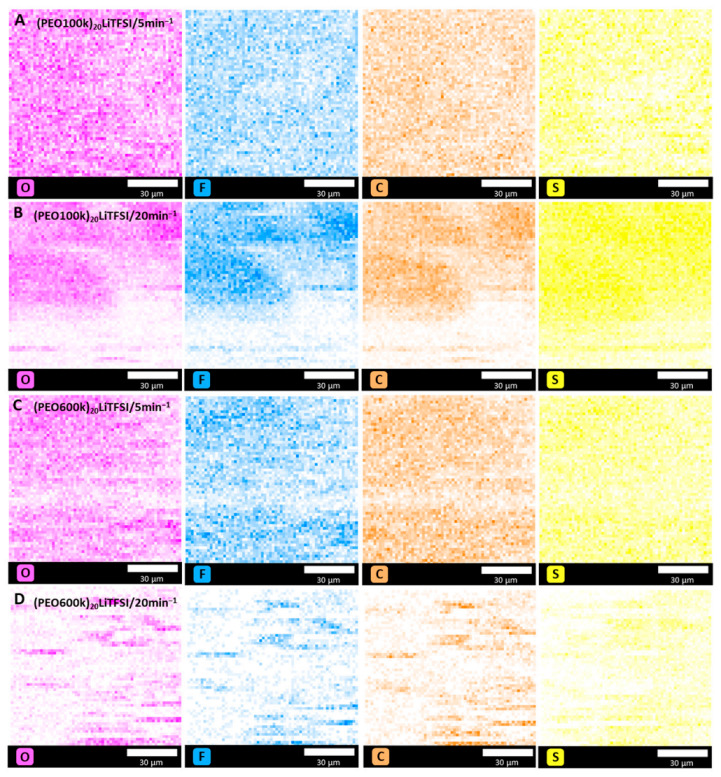
EDX mapping of the elements oxygen (O), fluorine (F), carbon (C) and sulfur (S) of kneaded (PEO100k)_20_LiTFSI (**A**,**B**) and (PEO600k)_20_LiTFSI (**C**,**D**) at 100 °C.

Figure 8 depicts the EDX mapping of the kneaded (PEO100k)_20_LiTFSI (A and B) and (PEO600k)_20_LiTFSI (C and D) at 100 °C and at kneading speeds of 5 and 20 min^−1^. The mapping shows that, for all kneaded electrolytes, LiTFSI was well distributed within the polymer matrix, characterized by the detection of fluorine (F) and sulfur (S). However, the distribution showed dependence on the applied kneading speed and the molecular weight. With increasing kneading speed, the distribution became less homogeneous. The same dependence can be observed with increasing molecular weight. The (PEO100k)_20_LiTFSI electrolyte showed the best and most homogeneous LiTFSI distribution for a kneading speed of 5 min^−1^.

### 3.5. GPC Measurements

Table 2 shows the number average molecular weight, M_n_, and the polydispersity, d, for the kneaded PEO and (PEO)_20_LiTFSI at 100 °C with kneading speeds of 5 and 20 min^−1^. The M_n_ of the kneaded PEO100k showed no significant dependence on the applied kneading speed. The decrease in the polydispersity from 5.61 at a low kneading speed to 5.32 at a high kneading speed implies a decrease in the molecular weight of the sample kneaded at 20 min^−1^. This was not detected in the kneading curve as a loss of torque (Figure 2). The decrease in d while the M_n_ was constant indicates that only the long polymer chains are shortened by chain scission due to mechanical shear. The same dependence can be seen in the GPC results for PEO600k. In comparison, PEO600k shows overall lower values for d than PEO100k. This indicates a narrower molecular weight distribution of PEO600k.

Comparing the results of M_n_ of neat PEO100k with M_n_ of (PEO100k)_20_LiTFSI, it is noticeable that the addition of LiTFSI shifted the M_n_ to lower values. This implies that all chain lengths were affected by chain scission during the process. The correlation between the applied screw speed and d was not further affected by LiTFSI. Considering the significant decrease in the M_n_ for (PEO600k)_20_LiTFSI from 168,700 to 90,800 at a kneading speed of 20 min^−1^, it is obvious that the addition of LiTFSI amplified the degradation of long polymer chains.

## 4. Conclusions

In this work, the influence of process parameters, molecular weight, and the addition of conductive salt on the thermal processing behavior of PEO was investigated. The results clearly highlight that care must be taken in the thermal processing of PEO. Preliminary DSC measurements show that T_m_ of PEO600k is shifted to a higher temperature due to larger crystals within the polymer matrix. The addition of the conductive salt provoked a reduction in the crystallinity of PEO and affected the amorphous region by shifting the glass transition temperature to higher temperatures for both polymer electrolytes.

Kneading experiments were used to investigate the thermal stability and kneading behavior of PEO during thermal processing, which is a combination of thermal and mechanical load. For a better evaluation of the kneading results, GPC measurements, a microscopic observation, and an EDX analysis of the kneaded samples were performed. Overall, PEO100k behaved more stably during the kneading time, showing the most homogeneous LiTFSI distribution at the lowest kneading speed. PEO600k was more sensitive to thermal and mechanical treatments. Degradation was observed due to self-heating and shear effects. LiTFSI acted as a plasticizer. However, the GPC results show that for both PEO samples, degradation occurred simultaneously with a softening of the polymer due to the addition of LiTFSI. Based on this knowledge, the observation of the torque alone is not meaningful enough to make a statement about the mechanical stability of PEO during processing. The decrease in torque is an indication of both the softening of the material due to the decoiling of the polymer chain, and polymer degradation due to the chain scission of long polymer chains. Thus, LiTFSI has two effects on the thermal processing of PEO. In addition to the plasticizing effect, LiTFSI also intensified the degradation of long chains of PEO. As a result, PEO600k was significantly more sensitive to and unstable after the addition of LiTFSI during thermal processing than PEO100k. Therefore, PEO100k is preferred for the thermal production of PEO-based solid electrolytes.

## Figures and Tables

**Table 1:** DSC data of neat-PEO and PEO_20_LiTFSI electrolytes.

Material	Glass Transition Temperature, T_g_	Melting Temperature, T_m_	Enthalpy of Melting, ΔH_m_	Crystallinity, K
PEO100k	−54 °C	61 °C	142 J g^−1^	70%
(PEO100k)_20_LiTFSI	−40 °C	61 °C	57 J g^−1^	28%
PEO600k	−55 °C	65 °C	142 J g^−1^	70%
(PEO600k)_20_LiTFSI	−34 °C	64 °C	56 J g^−1^	28%

**Table 2:** GPC results of kneaded neat-PEO and PEO_20_LiTFSI electrolytes.

	PEO100k				PEO600k			
	5 min^−1^		20 min^−1^		5 min^−1^		20 min^−1^	
	M_n_ (g mol^−1^)	d	M_n_ (g mol^−1^)	d	M_n_ (g mol^−1^)	d	M_n_ (g mol^−1^)	d
Kneaded	26,300	5.61	26,500	5.32	159,800	3.47	168,700	3.16
+LiTFSI	21,600	5.67	21,500	4.52	159,800	2.58	90,800	2.42

## Data Availability

The data presented in this study are available within this the article.
